# The Detection of SARS-CoV2 Antigen in Wastewater Using an Automated Chemiluminescence Enzyme Immunoassay

**DOI:** 10.3390/ijerph19137783

**Published:** 2022-06-24

**Authors:** Supranee Thongpradit, Somsak Prasongtanakij, Supanart Srisala, Suwannee Chanprasertyothin, Ekawat Pasomsub, Boonsong Ongphiphadhanakul

**Affiliations:** 1 Research Center, Faculty of Medicine Ramathibodi Hospital, Mahidol University, Bangkok 10400, Thailand; supranee.ton@mahidol.ac.th (S.T.); supanart.sri@mahidol.ac.th (S.S.); suwannee.cha@mahidol.ac.th (S.C.); bsong.ong@gmail.com (B.O.); 2 Department of Pathology, Faculty of Medicine Ramathibodi Hospital, Mahidol University, Bangkok 10400, Thailand; ekawat.pas@mahidol.ac.th; 3 Department of Medicine, Faculty of Medicine Ramathibodi Hospital, Mahidol University, Bangkok 10400, Thailand

**Keywords:** COVID-19, wastewater, CLEIA, SARS-CoV-2 antigen, RT-qPCR

## Abstract

The SARS-CoV-2 virus, which is driving the current COVID-19 epidemic, has been detected in wastewater and is being utilized as a surveillance tool to establish an early warning system to aid in the management and prevention of future pandemics. qPCR is the method usually used to detect SARS-CoV-2 in wastewater. There has been no study using an immunoassay that is less laboratory-intensive than qPCR with a shorter turnaround time. Therefore, we aimed to evaluate the performance of an automated chemiluminescence enzyme immunoassay (CLEIA) for SARS-CoV-2 antigen in wastewater. The CLEIA assay achieved 100% sensitivity and 66.7% specificity in a field-captured wastewater sample compared to the gold standard RT-qPCR. Our early findings suggest that the SARS-CoV-2 antigen can be identified in wastewater samples using an automated CLEIA, reducing the turnaround time and improving the performance of SARS-CoV-2 wastewater monitoring during the pandemic.

## 1. Introduction

Coronavirus disease 2019 (COVID-19), caused by the severe acute respiratory syndrome coronavirus 2 (SARS-CoV-2), became a global pandemic in a relatively short amount of time, with extensive repercussions on health, the economy, and society.

During the COVID-19 public health emergency, several publications revealed the presence of SARS-CoV-2 RNA in stools from COVID-19 patients and the occurrence of SARS-CoV-2 in wastewaters globally. As an outcome, during the ongoing COVID-19 pandemic, wastewater surveillance for SARS-CoV-2 has proven to be a practical and sensitive tool for assessing the prevalence and tracking the transmission of SARS-CoV-2 in diverse nations and scenarios. It was demonstrated that SARS-CoV-2 might be identified in untreated and treated wastewater [1,2,3,4]. Similar findings from further research in various geographic locations have led the United States Center for Disease Control to advise SARS-CoV-2 wastewater surveillance [5].

The current gold standard for COVID-19 microbiological diagnosis is the identification of SARS-CoV-2 genetic targets in various types of specimens utilizing the molecular real-time reverse transcription–polymerase chain reaction (RT-PCR) [6], including wastewater which has been demonstrated as a possible source of specimen for surveilling and monitoring SARS-CoV-2 outbreaks [5]. Despite its sensitivity, RT-qPCR in wastewater surveillance of SARS-CoV-2 is time-consuming and labor-intensive, requiring specialized laboratory equipment and skilled technicians [6,7]. Recently, a sensitive and comfortable assay for the detection of SARS-CoV-2 using chemiluminescence enzyme immunoassay (CLEIA) has been designed to detect SARS-CoV-2 antigens, capable of detecting and quantifying nucleocapsid protein (NP) of SARS-CoV-2 in both nasopharyngeal swabs and saliva samples [8,9,10]. In Japan, the CLEIA assay for identifying SARS-CoV-2 nucleocapsid (N) protein has been utilized since June 2020, and a positive antigen test result is adequate to definitely diagnose COVID-19 without a PCR [11], which is instead mandatory in European countries to confirm positive antigen results [12]. Furthermore, because to their 35 min test result turnaround, the CLEIA assay is widely employed in Japanese airports for COVID-19 screening. For community and population screening, Gill et al. have used the CLEIA assay to detect SARS-CoV-2 nucleocapsid protein in nasopharyngeal swabs [13].

The present researchers focused on the potential role of an automated CLEIA assessment in wastewater. Towards that end, we used an automated CLEIA assay to evaluate the quality of SARS-CoV-2 antigen detection. For that purpose, we compared the antigen results of the CLEIA assay with the results of an RT-qPCR assay targeting SARS-CoV-2 genomic RNA.

## 2. Materials and Methods

**Figure 1:**
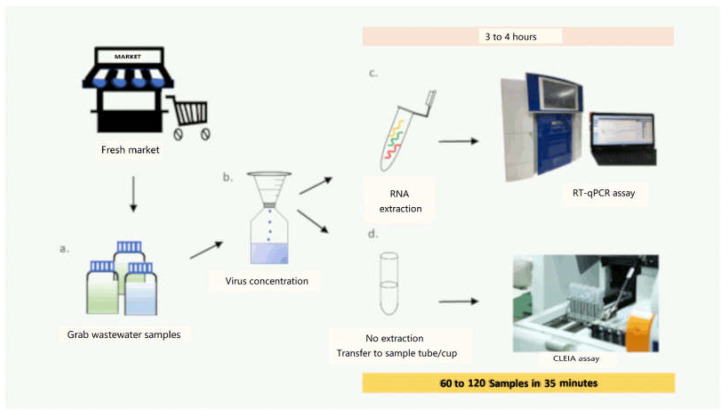
SARS-CoV-2 detection in grab wastewater samples from different fresh markets using RT-qPCR and CLEIA: (**a**) grab wastewater samples were taken from 14 different fresh markets and collected in clean plastic bottle; (**b**) concentration of SARS-CoV-2 virus in wastewater samples using a mixed cellulose ester membrane filter (pore size, 0.45  µm; diameter, 47  mm); (**c**) virus RNA was extracted directly from the filtered membrane and SAR-CoV-2 virus was detected and quantified by RT-qPCR; (**d**) detection of the SAR-CoV-2 virus antigen in concentrated wastewater using CLEIA.

Research Methodology to determine SARS-CoV-2 in grab wastewater samples shown in Figure 1.

### 2.1. The Limit of Detection (LOD)

The limit of detection (LOD) of the CLEIA was determined based on the RT-qPCR assays as a reference control of the ten-fold serial spike dilutions of inactivated SARS-CoV-2-infected cells ranging from 10^7^ to 10^1^. As a control, autoclaved pool wastewater was used. The tests were performed in three duplicates.

#### 2.1.1. Sample Preparation and Concentration

A subsample grab sample (100–400 mL) was centrifuged at 3000× *g* for 10 min. The supernatant was then filtered through a mixed cellulose ester membrane filter (pore size, 0.45 m; diameter, 47 mm; GE Healthcare, Chicago, IL, USA) attached to a consumable MillicupTM-FLEX filtration unit (Merck Ltd., Darmstadt, Germany), and the assembly filtration equipment was vacuumed until the filtration was complete. The membrane filter was placed in a sterile 5 mL tube upon removal. Afterward, 1 mL of DNA/RNA ShieldTM and 0.1 g of ZR BashingBead (Zymo Research, Sigma, Irvine, CA, USA) were added to each tube. Then, the prepared solution was then mixed 10 times with a vortex mixer at an approaching speed (60 s each). Following this, 400 μL of the solution was transferred into a new nuclease-free tube and maintained at −80 °C until the assay was finished.

#### 2.1.2. SARS-CoV-2 Virus Detection and Quantification by RT-qPCR

RNA extract and RT-qPCR experiments targeting ORF1ab, spike regions (S), and the nucleocapsid (N) of the SARS-CoV-2 viral RNA were conducted and evaluated, as described in our previous work [14]. The detection of SARS-CoV-2 was classified as positive if it contained positive findings for two or more SARSCoV-2 target genes, designated at a cycle threshold (Ct) smaller than 37.

#### 2.1.3. Detection of SARS-CoV-2 Antigen

A specific chemiluminescence-based immunoassay method was analyzed on the Lumipulse G1200 automated immunoassay analyzer (Fujirebio) [9]. The antigen cutoff concentration for nasopharyngeal samples (1.34 pg/mL) recommended by the manufacturer was used to classify the results as positive for SARS-CoV-2 detection.

### 2.2. The Field Performance of the SARS-CoV-2 CLEIA

To assess the SARS-CoV-2 CLEIA’s field performance, 14 grab wastewater specimens were collected during an outbreak in Bangkok and neighboring areas from three fresh markets, two of which had confirmed COVID-19 cases (PP and SC markets), whereas the third (RS market) did not. Grab samples of wastewater were collected in cleaned bottles from each market’s wastewater and delivered to the laboratory on ice, where they were kept at 4 °C until further analysis.

## 3. Results

We determined the analytical capability of the CLEIA assay. As shown in Table 1, the assay was capable of detecting SARS-CoV-2 in wastewater down to the 10^4^ spike cell, whereas RT-qPCR was able to detect SARS-CoV-2 in wastewater down to the 10^2^ spike cell.

Of the 14 grabbed wastewater samples from three fresh markets, there were 8 positive samples for SARS-CoV-2 (Ct-value < 37) and 6 negative samples (Ct-value ≥ 37), as determined by RT-qPCR. All positive RT-qPCR samples tested positive in the CLEIA assay, whereas two out of six negative RT-qPCR samples tested positive and the rest tested negative. As indicated in Table 2, in comparison to RT-qPCR, the CLEIA demonstrated four true negatives (samples P5, P6, S3, and R1) and two false positives (samples RS2 and RS3). The remaining samples were true positive. There was no indication of a false negative. Taken together, the CLEIA assay had sensitivity and specificity scores of 100% and 66.7%, respectively.

## 4. Discussion

To our knowledge, this is the first report on the detection of SARS-CoV2 in wastewater using the CLEIA assay. A suitable SAR-CoV-2 antigen cutoff level for the CLEIA in wastewater is missing. We used the previous cutoff value of 1.34 pg/mL, which was recommended by the manufacturer, suggested by the study on the nasal pharyngeal swab sample which utilized the corresponding test instrument [9]. In contrast, the manufacturer’s current diagnostic cutoffs for saliva specimens were 0.67 pg/mL [15]. As an outcome, it is possible that the cutoff value for wastewater samples may be different and needs to be more appropriately explored.

Using RT-PCR as the best available comparator method, CLEIA generally has a lower sensitivity, but it has several benefits over PCR, including quickness, cheap cost, ease of availability, and performance convenience [16]. In this study, we showed that the CLEIA assay had a lower limit of detection than RT-qPCR in serial dilutions experiments. It performed reasonably well in detecting SARS-CoV-2 in grabbed wastewater samples during an outbreak with 100% sensitivity and 66.7% specificity. The lower specificity may be due to non-specific interference from materials in wastewater, which could be diminished by dilution [17]. In terms of efficiency, the automated CLEIA assay can handle 60–120 samples in 35 min, significantly reducing the turnaround time. The number of false-negative and false-positive results driven by these metrics was zero (among eight RT-PCR-positive results) and two (among six RT-PCR-negative results), respectively.

The performance of the CLEIA assay used in the present study for wastewater was in line with previous results using nasopharyngeal samples. Hirosu et al. established the technique’s accuracy. The antigen assay detected the SARS-CoV-2 antigen in nasopharyngeal samples with a viral load more than 100 copies per test, and the results were completely compatible with RT-qPCR results. The sensitivity decreased to 85% in samples with a viral load of less than 100 copies per test [18]. Another study found that the antigen assay exhibited sensitivity of 92.3% (95% CI: 89.2–92.3%) and specificity of 100% (95% CI: 95.5–100%), compared with RT-PCR [19]. Another report by Menchinelli et al. showed that the CLEIA assay was highly sensitive in samples with low RT-qPCR Ct values in clinical samples, i.e., 93 to 100% with samples that displayed RT-qPCR Ct values below 25–30. Furthermore, a significant drop to 47.9 and 42.1 % was recorded with Ct values of 30–35 and 35–40, respectively [9]. Using the CLEIA test, Ishii et al. demonstrated great sensitivity for both saliva (96.9%) and nasopharyngeal swabs (99.6%) [20]. Sberna et al. demonstrated strong and highly significant correlation between the CLEIA and the RT-PCR Ct values (RdRp gene) to determine SARS-CoV-2 on 513 nasopharyngeal swabs. The sensitivity of CLEIA was >95% for samples with Ct < 30 [21].

Currently, SARS-CoV-2 genome mutations have been discovered, as well as a number of important variations, including multiple variants of concern (VOC), such as the Delta and Omicron variants. As a corollary, the SARS-CoV-2 genetic variants may pose challenges in identifying new positives. Remarkably, Yin et al. reported that no variation of viral effect was identified in 93 strong positive samples [22], whereas Osterman et al. demonstrated that the CLEIA assay could carry efficacy to determine alpha (B.1.1.7) or beta (B.1.351) [23]. Similar with Gandolfo et al., CLEIA can identify and quantify SARS-CoV-2 nucleocapsid protein in saliva or nasopharyngeal swabs. Furthermore, the assay could identify the variant type and exhibit amino acid substitutions inside or close to the functional N antigenic epitope, including B.1.1.7, B.1.1.34, B.1.1.420, B.1.177.75, B.1.258, B.1.351, and P.1 [24]. These findings show that the protein area identified by the CLEIA assay is unaffected by mutations, allowing the identification of all variations investigated in this work. 

In addition, reverse transcription loop-mediated isothermal amplification (RT-LAMP) has shown promise and may provide a quick and low-cost technique for observing SARS-CoV-2 in wastewater, as well as amplification within 35 min. Unfortunately, there are limitations to the test’s quantification and limit of detection [25]. The GeneXpert system reported by Daidle et al. is another fully automated assay used to determine SARS-CoV-2 in wastewater within 37 min. However, sensitivity is acceptable at concentrations over 32 copy/mL in wastewater. Without concentration, the findings should be considered qualitative [26].

## 5. Conclusions

Our preliminary results suggest that the SARS-CoV-2 antigen can be well detected in wastewater samples using an automated CLEIA, which can minimize turnaround time and increase the performance of SARS-CoV-2 wastewater monitoring during the pandemic.

## Figures and Tables

**Table 1:** The limit of detection (LOD) of the CLEIA assay was determined by comparing it to RT-qPCR assays as a reference control.

Spike Cell	CLEIA		RT-qPCR			
	Antigen Concentrations (pg/mL)	Interpretation	N (Mean Ct ± SD)	ORF1ab (Mean Ct ± SD)	S (Mean Ct ± SD)	Interpretation
10^7^	2399.67	Positive	33.04 ± 0.65	33.02 ± 0.45	23.04 ± 3.56	Positive
10^6^	396.08	Positive	23.25 ± 0.02	22.81 ± 0.13	23.45 ± 1.86	Positive
10^5^	37.56	Positive	26.16 ± 0.08	25.97 ± 0.17	29.39 ± 2.93	Positive
10^4^	3.63	Positive	29.34 ± 0.26	28.84 ± 0.19	26.84 ± 1.66	Positive
10^3^	0.39	Negative	32.52 ± 0.24	31.56 ± 0.39	23.96 ± 1.97	Positive
10^2^	0.01	Negative	36.05 ± 0.96	29.56 ± 0.00	24.40 ± 2.48	Positive
10^1^	0.01	Negative	37.27 ± 1.44	31.97 ± 5.79	24.19 ± 6.02	Inconclusive
No spike	UD	Negative	UD	UD	UD	Negative

Note: UD—undetermined. Positive results were defined as antigen cutoffs greater than 1.34 pg/mL in the CLEIA assay and cycle threshold (Ct) <  37, for two or more SARS-CoV-2 target genes in the RT-qPCR assay.

**Table 2:** Detection of SARS-CoV-2 in grabbed wastewater from 3 markets (PP, SC, and RS) using the RT-qPCR and CLEIA assays.

Sample	RT-qPCR (Ct)				CLEIA	
	N	ORF1ab	S	Interpretation	Antigen Concentration (pg/mL)	Interpretation
PP1	34.21	31.89	33.98	Positive	17.2	Positive
PP2	32.24	30.94	34.85	Positive	9.68	Positive
PP3	UD	33.35	UD	Positive	30.78	Positive
PP4	UD	33.83	36.97	Positive	11.76	Positive
PP5	UD	UD	UD	Negative	0.39	Negative
PP6	UD	UD	UD	Negative	0.44	Negative
PP7	33.39	30.46	31.32	Positive	1.9	Positive
PP8	33.96	31.77	33.92	Positive	38.46	Positive
SC1	UD	36.51	UD	Positive	13.5	Positive
SC2	36.41	34.81	UD	Positive	8.52	Positive
SC3	UD	UD	UD	Negative	0.27	Negative
RS1	UD	UD	UD	Negative	0.9	Negative
RS2	UD	UD	UD	Negative	7.19	Positive
RS3	UD	UD	UD	Negative	1.83	Positive

Note: UD—undetermined. Positive results were defined as antigen cutoffs greater than 1.34 pg/mL in the CLEIA assay and cycle threshold (Ct)  <  37, for two or more SARS-CoV-2 target genes in the RT-qPCR assay.
